# Analysis of G-quadruplexes upstream of herpesvirus miRNAs: evidence of G-quadruplex mediated regulation of KSHV miR-K12–1-9,11 cluster and HCMV miR-US33

**DOI:** 10.1186/s12860-020-00306-w

**Published:** 2020-09-24

**Authors:** Shivani Kumar, Divya Choudhary, Anupam Patra, Neel Sarovar Bhavesh, Perumal Vivekanandan

**Affiliations:** 1 Kusuma School of Biological Sciences, Indian Institute of Technology Delhi, New Delhi, India; 2 Department of Chemical Engineering, Indian Institute of Technology Delhi, New Delhi, India; 3 Transcription Regulation Group, International Centre for Genetic Engineering and Biotechnology, New Delhi, India

**Keywords:** Herpesvirus, G-quadruplexes, MiRNA, Kaposi's sarcoma-associated Herpesvirus, Human Cytomegalovirus

## Abstract

**Background:**

G-quadruplexes regulate gene expression, recombination, packaging and latency in herpesviruses. Herpesvirus-encoded miRNAs have been linked to important biological functions. The presence and the biological role of G-quadruplexes have not been studied in the regulatory regions of virus miRNA. We hypothesized that herpesvirus-encoded miRNAs are regulated by G-quadruplexes in their promoters.

**Results:**

We analyzed the 1 kb regulatory regions of all herpesvirus-encoded miRNAs for the presence of putative quadruplex-forming sequences (PQS). Over two-third (67%) of the regulatory regions of herpesvirus miRNAs had atleast 1 PQS. The 200 bp region of the promoter proximal to herpesvirus miRNA is particularly enriched for PQS. We chose to study the G-quadruplex motifs in the promoters of miR-K12 cluster in Kaposi's sarcoma-associated Herpesvirus (KSHV miR-K12–1-9,11) and the miR-US33 encoded by Human Cytomegalovirus (HCMV miR-US33). Biophysical characterization indicates that the G-quadruplex motifs in the promoters of the KSHV miR-K12 cluster and the HCMV miR-US33 form stable intramolecular G-quadruplexes in vitro. Mutations disrupting the G-quadruplex motif in the promoter of the KSHV miR-K12 cluster significantly inhibits promoter activity, while those disrupting the motif in the promoter of HCMV miR-US33 significantly enhance the promoter activity as compared to that of the respective wild-type promoter. Similarly, the addition of G-quadruplex binding ligands resulted in the modulation of promoter activity of the wild-type promoters (with intact G-quadruplex) but not the mutant promoters (containing quadruplex-disrupting mutations).

**Conclusion:**

Our findings highlight previously unknown mechanisms of regulation of virus-encoded miRNA and also shed light on new roles for G-quadruplexes in herpesvirus biology.

## Background

G-rich nucleic acids are known to form G-quadruplexes which are secondary structures composed of two or more G-tetrads stacked upon one another and the four Gs of the tetrad are bound by Hoogsteen hydrogen bonding [1–3]. In the human genome, G-quadruplexes are enriched in promoters of oncogenes such as *c-myc, bcl2, RET, c-kit2, k-RAS, MET,* and *YY1* [4–10]. These DNA secondary structures are capable of regulating crucial cellular processes including transcriptional and translational regulation, telomere maintenance and recombination [11–14]. Recent studies have shed light on the biological significance of G-quadruplexes in viruses including their role in regulating transcription, virus latency, virus recombination, virus packaging and replication [15–28].

MicroRNAs (miRNAs) are short non-coding RNAs (~ 22 nucleotides long) that regulate gene expression. MiRNAs can bind to the 3′ untranslated region (UTR), the 5′ UTR, or the coding region of a gene and induce mRNA degradation or translational repression [29–31]. MiRNAs regulate many cellular processes, including cellular proliferation and differentiation, cell death, oncogenesis, and defense mechanisms [32, 33].

To date, more than 250 organisms are known to express miRNAs [34]. According to miRBase database [34], a total of 31 viruses encode > 500 mature miRNA species; this includes a large number of miRNAs that are encoded by herpesviruses. Herpesviruses are ubiquitous large enveloped double-stranded DNA viruses that infect humans and animals, and are capable of establishing latency which allows them to cause chronic infections in the host. Herpesviruses express an array of distinct miRNAs that are known to play a critical role in maintaining viral latency [32, 35, 36]. Many eukaryotic genomes have been analyzed for putative promoters in the upstream regulatory regions of miRNAs [37–40]. A large subset of intronic miRNAs were found to possess regulatory elements proximal to the miRNA coding regions [38]. Monteys et al. (2010) indicated the presence of transcription regulatory elements in the 5 kb region upstream of the intronic miRNA species with majority localized within the 0-1 kb region [41, 42]. Only few herpesvirus miRNA promoters are well characterized in literature [43–46]. MiRNAs encoded by human cytomegalovirus (HCMV) are found interspersed within the viral genome and hence their expression is regulated via promoters of adjacent viral genes [47, 48]. Epstein Barr virus (EBV) encodes for 2 distinct groups/clusters of miRNAs (EBV BART and BHRF1 miRNAs) which are localized in the intronic regions of longer transcripts and therefore share a common promoter with BART and BHRF1 transcripts respectively [32, 49, 50].

The presence of G-quadruplex structures in the regulatory regions of virus encoded miRNAs and their effect on virus miRNA expression has not been investigated. Herpesviruses account for over 90% of all virus encoded miRNAs. We therefore sought to analyze the upstream regulatory regions of all known herpesvirus miRNAs for putative G-quadruplex forming sequences (PQS). About 67% of the miRNAs analyzed possess at least 1 PQS motif in their upstream regulatory regions. We performed extensive biophysical characterization and investigated the biological role of the PQS in the regulatory region of miRNAs encoded by Kaposi's sarcoma-associated Herpesvirus (KSHV) and Human Cytomegalovirus (HCMV). KSHV encoded miRNAs (miR-K12–1-9,11) are localized in the KSHV latency associated region (KLAR) interspersed between latency related genes (i.e. LANA, vCyclin, vFLIP, and kaposin K12 genes) [51, 52]. These viral miRNAs are known to target an array of host as well as virus encoded genes. KSHV miR-K12–7 and 9 repress KSHV RTA (ORF50) transcription [52, 53]; miR-K12–1 targets IκBα, (NF-κB inhibitor) [54]; miR-K12–5 and 9 downregulates the Bcl-2-associated factor (BCLAF1) [55]; miR-K12–11 targets IKKε, a signalling intermediate [56]. A recent finding shows that KSHV miR-K12–6-5p mimics the human miR-15/16 family of miRNAs and share mRNA targets [57]. Although, KSHV miRNA cluster was earlier reported to be expressed via a distal promoter present upstream of LANA [51], Pearce et al. (2005) indicates presence of a proximal promoter coinciding with v-Cyclin and v-FLIP transcription units [58]. HCMV miR-US33 is encoded in conjunction with *US33* gene and hence, share a common promoter [47, 59]. HCMV miR-US33 represses viral DNA replication in the host by binding to the 3′ UTR of host syntaxin mRNA [60]. HMCV miR-US33 is also reported to target viral *US29* gene [48].

Our findings suggest that the PQS identified in the regulatory regions of herpesviruses do form G-quadruplex structures and can contribute significantly to transcriptional regulation of virus encoded miRNA. This work highlights the existence of a novel transcriptional regulatory mechanism that modulates expression of hepervirus miRNAs.

## Results

### PQS motifs are abundant in putative herpesvirus miRNA promoters

**Fig. 1:**
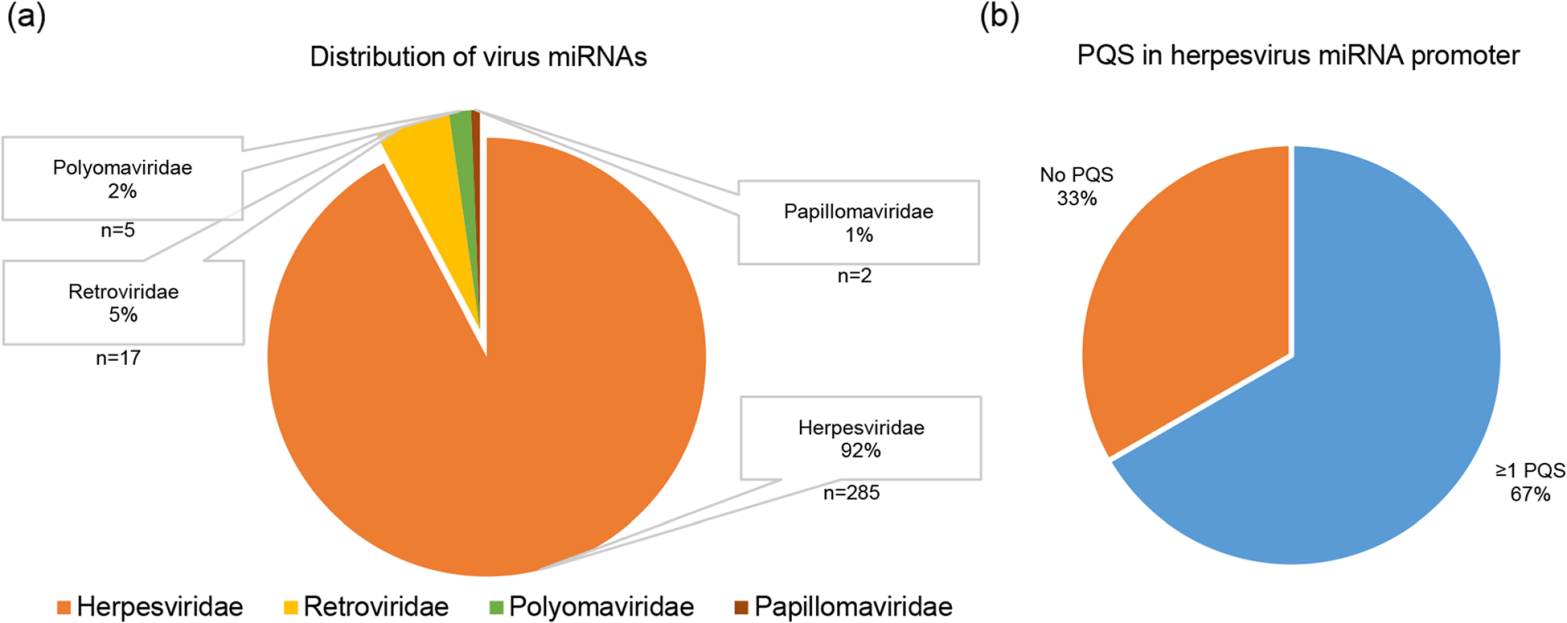
Virus miRNAs and distribution of putative quadruplex forming sequences (PQS). (**a**) Distribution of virus-encoded miRNAs across virus families; herpesviruses account for more than 90% of all virus-encoded miRNAs. (**b**) About 67% of all putative herpesvirus miRNA promoters were found to possess at least 1 putative quadruplex forming sequence (PQS) motif as analyzed by Quadparser [61]

The miRBase database (version 22.1) has released 530 viral miRNA species [34]. Of these, the vast majority (92%) of miRNAs are encoded by herpesviruses (Fig. 1a). It is widely accepted that putative regulatory elements of herpesvirus genes are primarily located in the 1 kilobase (kb) upstream region [23]. Therefore, we analyzed all available herpesvirus precursor miRNAs for putative quadruplex forming motifs (PQS motifs) in the 1 kb upstream regulatory region. Quadparser [61] was used to identify PQS motifs and about 67% of these precursors contain at least 1 PQS motif in their regulatory elements (Fig. 1b).

**Fig. 2:**
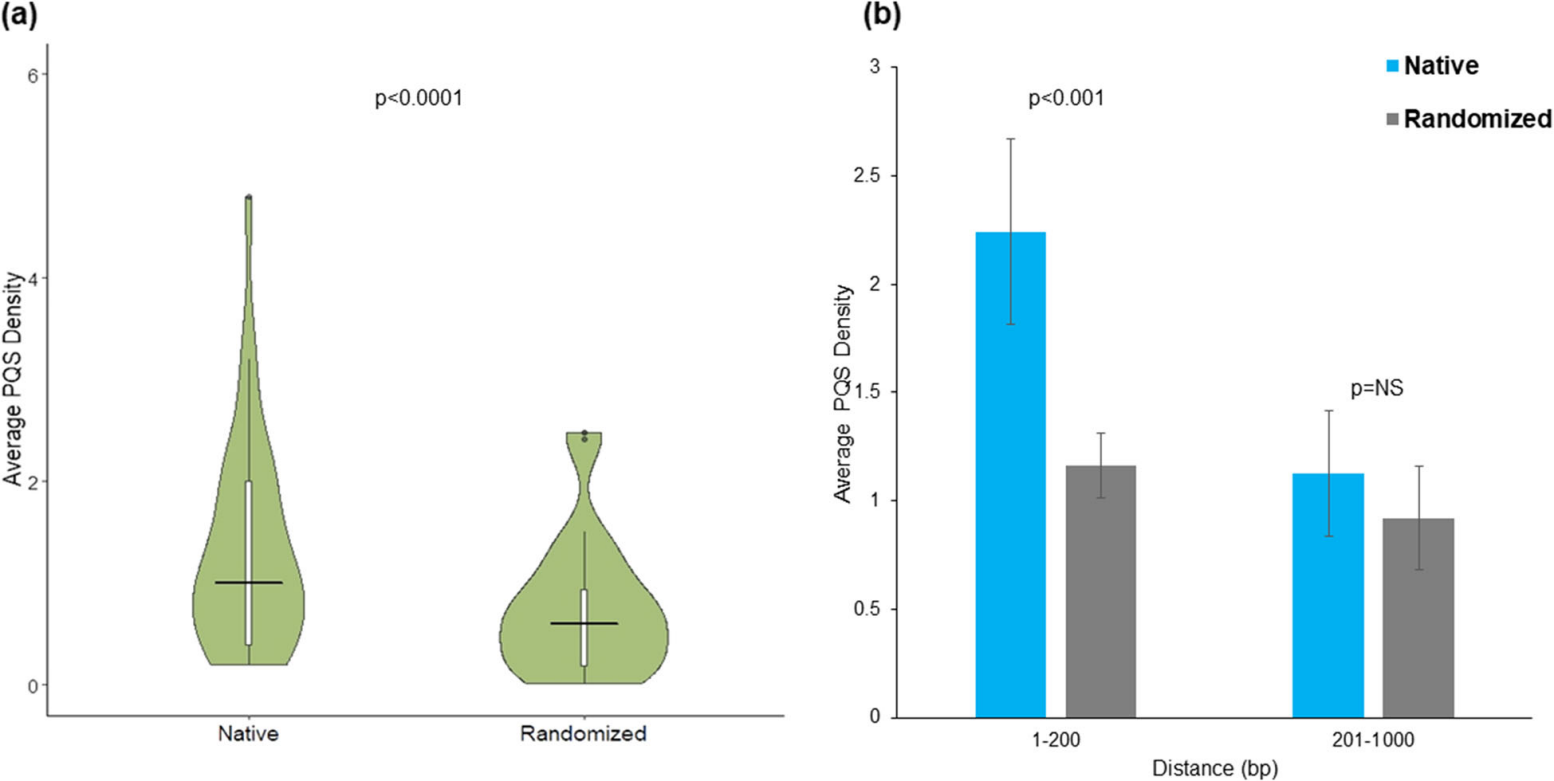
Enrichment of PQS in the putative promoters of herpesvirus miRNAs. (**a**) Violin plot shows the distribution of PQS in native vs randomized sequences. The horizontal black line in the middle of the plot represents the median values for the PQS density. (**b**) The average PQS densities of the native promoter sequences are significantly higher in the first 200 bp upstream of virus precursor miRNA as compared to that in randomized sequences. Also, the distribution of PQS within the 201–1000 bp window of the native and randomized datasets are comparable to each other. Statistical significance was calculated using Wilcoxon matched-pair signed rank test. *P* values less than 0.01 were considered significant. NS denotes not significant. The error bars represent the standard deviation within the two datasets

**Fig. 3:**
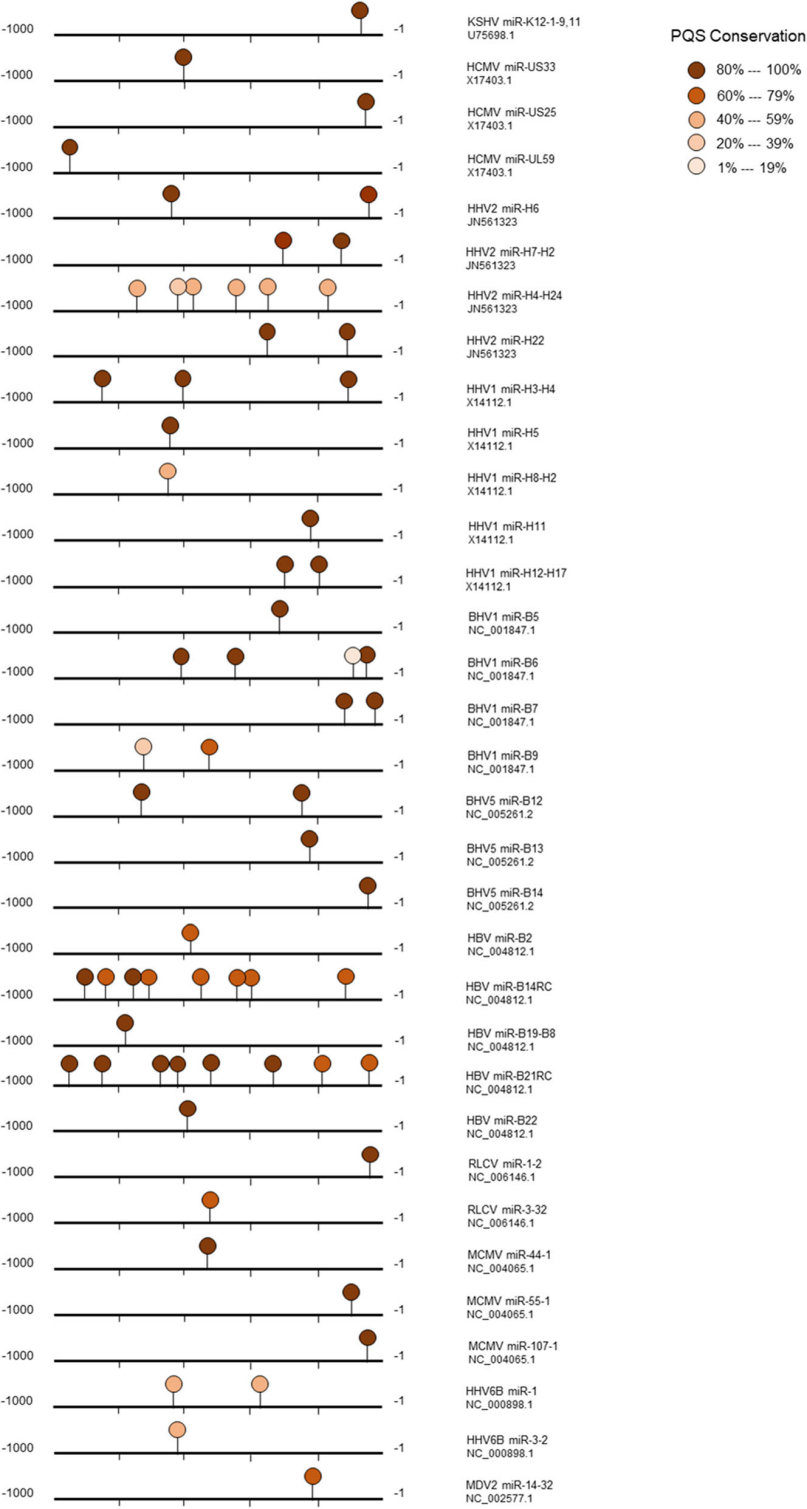
Conservation of PQS motifs upstream of herpesvirus miRNAs. All available full-length herpesvirus sequences were used for conservation analysis. The name of the virus miRNA and the GenBank accession number of the sequence used to depict the location of the PQS are listed adjacent to each sequence (*n* = 33). All viral strains analyzed are listed in Additional file 1; Table S4. Additional details about the conservation of PQS found in herpesvirus miRNA promoters is available in Additional file 1; Table S5

To determine whether the occurrence of PQS motifs within a herpesvirus miRNA regulatory element is a random feature that is merely a consequence of the nucleotide composition, we used a randomization approach. This analysis was performed on all herpesvirus miRNA regulatory sequences from five different strains for each virus. If five sequences were not available for a given virus, then all the available sequences were used. Each regulatory sequence was randomized five times without changing the overall nucleotide composition as described in the methods section; the PQS density (i.e. the number of PQS/kb) of the native and randomized sequences was calculated. The actual PQS density and the PQS density in the randomized sequences were plotted as shown in Fig. 2a. The results suggested that the PQS density in the regulatory regions (native) of herpesvirus miRNAs is much higher than that in the randomized regulatory regions [Median PQS density in regulatory region of miRNA (native) vs Median PQS density in randomized regulatory region of miRNA (randomized); Wilcoxon matched-pair signed rank test; *P* < 0.0001]. Interestingly, within the regulatory regions of herpesvirus miRNAs, PQS were significantly enriched within the first 200 base pairs (bp) from the precursor miRNAs compared to the rest of the promoter (i.e. 1–200 bp; Fig. 2b). This is in keeping with the significant enrichment of PQS densities in proximal regulatory regions of human promoters compared to the distal regions of the promoters [62, 63]. We also observed that the PQS motifs upstream of herpesvirus miRNAs were conserved across sequences for a given virus (Fig. 3).

### Biophysical characterization of GQ motifs flanking KSHV and HCMV miRNAs

The GQ motifs present upstream of KSHV miR-K12–1-9,11 cluster and HCMV miR-US33 were selected for further analysis because (a) the genomic DNA of KSHV and HCMV was readily available in our laboratory and (b) the putative promoters of miRNAs had at least 1 PQS motif each.

**Fig. 4:**
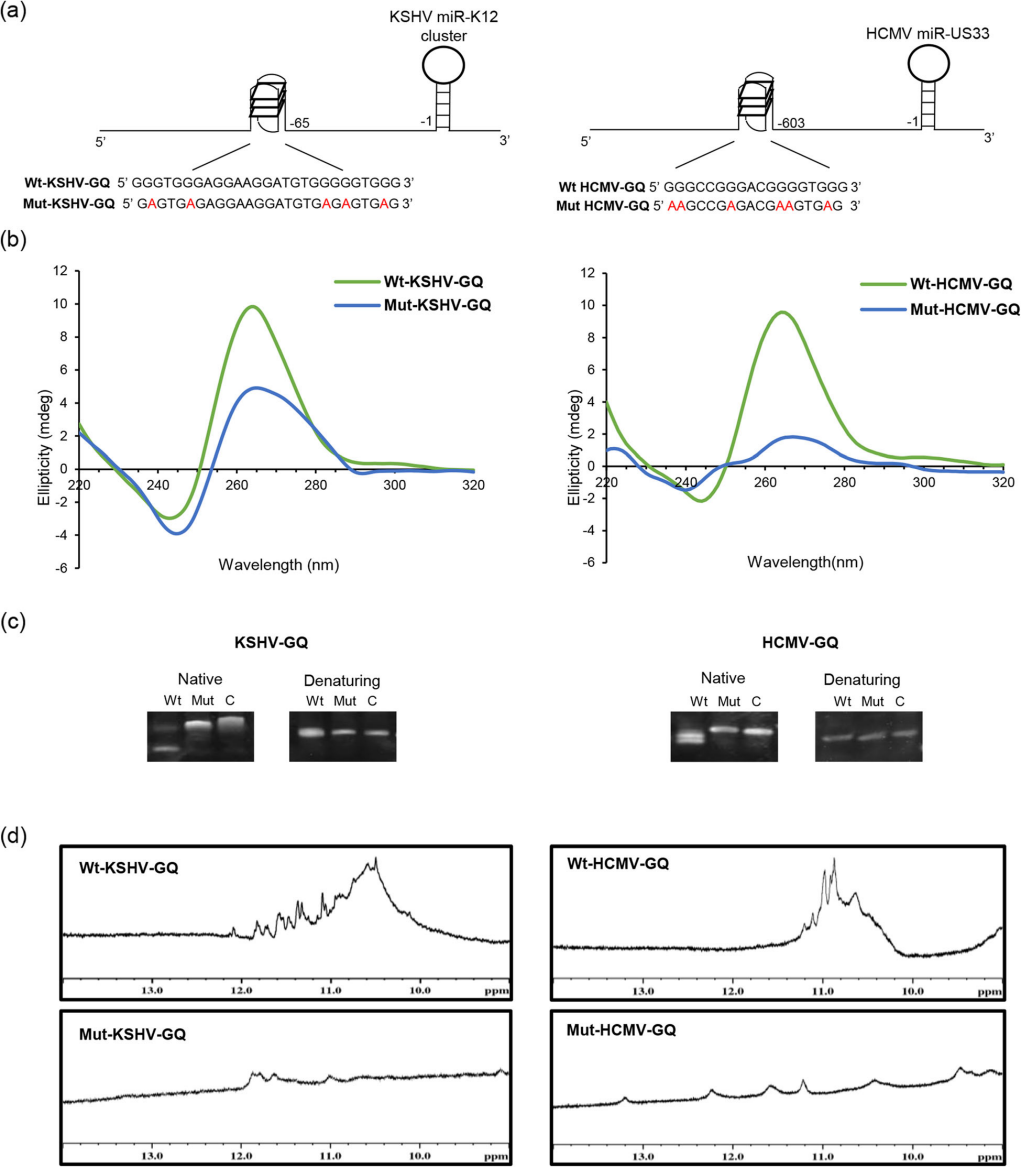
Biophysical analyses of KSHV-GQ and HCMV-GQ. (**a**) KSHV and HCMV PQS oligonucleotide sequences and their positions with respect to respective precursor miRNAs. The wild-type (Wt) oligonucleotide possesses the intact G-quadruplex motif while the mutant (Mut) oligonucleotide contains G-quadruplex disrupting mutations. (**b**) The CD spectra shows formation of parallel G-quadruplex structures for oligonucleotides Wt-KSHV-GQ and Wt-HCMV-GQ. (**c**) Native polyacrylamide gel electrophoresis indicates intramolecular G-quadruplex structure formation as depicted by higher mobility of both the wild-type oligonucleotides (Wt-KSHV-GQ and Wt-HCMV-GQ) compared to that of the mutant oligonucleotides (Mut-KSHV-GQ and Mut-HCMV-GQ) and C (length-matched controls: 27mer and 18mer sequences that do not form DNA secondary structures). On the other hand, denaturing polyacrylamide gel electrophoresis shows comparable migration rate for the wild-type, mutant and the length-matched controls in presence of 7 M urea (denaturant). (**d**) 1D ^1^H NMR spectra of Wt-KSHV-GQ and Wt-HCMV-GQ oligonucleotide show imino proton peaks in the range of 10.5–12 ppm; these peaks were not observed in case of mutants

To determine the topology of the GQs [64], CD spectra were recorded for the oligonucleotides corresponding to the PQS in the regulatory regions of KSHV miR-K12 cluster (Wt-KSHV-GQ) and HCMV miR-US33 (Wt-HCMV-GQ) and their respective mutants (details of the mutations disrupting the PQS are shown in Fig. 4a and in Additional file 1; Table S2). Both Wt-KSHV-GQ and Wt-HCMV-GQ adopt parallel G-quadruplex structure conformation with a positive peak around 260 nm and a negative peak around 240 nm. As expected the mutants of the two PQS (i.e. Mut-KSHV-GQ and Mut-HCMV-GQ) show disrupted quadruplex formation as indicated by their CD spectra (Fig. 4b). The mobility of Wt-KSHV-GQ and Wt-HCMV-GQ along with their respective mutants, was analyzed by comparing native and denatured polyacrylamide gel electrophoresis profiles. The wild type GQs migrated faster than the mutants and the control oligonucleotides, indicating that both the Wt-KSHV-GQ and Wt-HCMV-GQ fold into compact intramolecular structures (Fig. 4c).

**Fig. 5:**
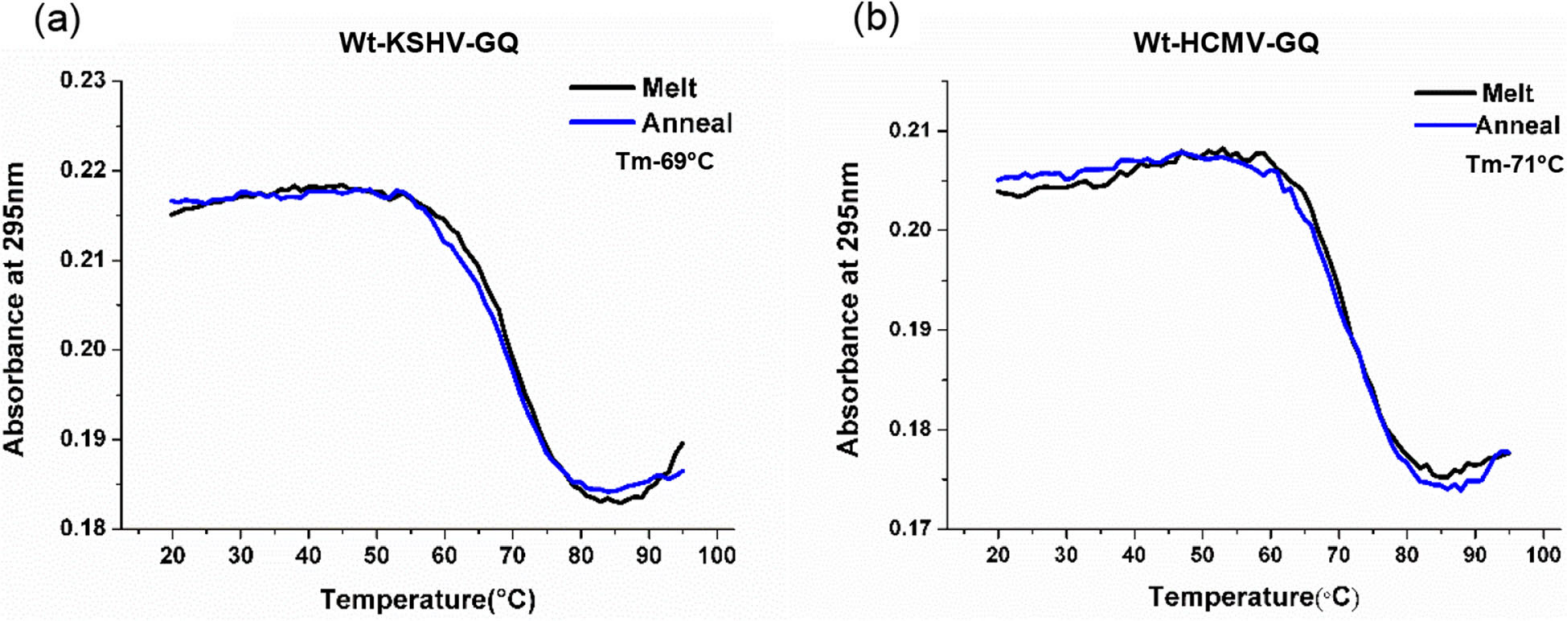
UV melting analysis. The melt curves were obtained for (**a**) Wt-KSHV-GQ and (**b**) Wt-HCMV-GQ oligonucleotides by monitoring UV absorbance at 295 nm as a function of temperature (20–93 °C). The superimposable curves are indicative of formation of intramolecular G-quadruplexes

The NMR spectra of both Wt-KSHV-GQ and Wt-HCMV-GQ showed that the G-imino protons involved in G-quadruplex formation exhibit a distinct proton chemical shift value of 10.5–12 ppm (Fig. 4d). Furthermore, upon G-quadruplex melting, the UV absorbance at 295 nm declines, leading to a hypochromic shift. The melting and annealing curves could be superposed on one another. This phenomenon suggests the formation of intramolecular G-quadruplexes [65] (Fig. 5a and b). Taken together, our biophysical analyses suggest that both Wt-KSHV-GQ and Wt-HCMV-GQ could fold into intramolecular parallel G-quadruplex structures in vitro.

### Stability studies of viral GQs in the presence of TMPyP4 and Pyridostatin

**Fig. 6:**
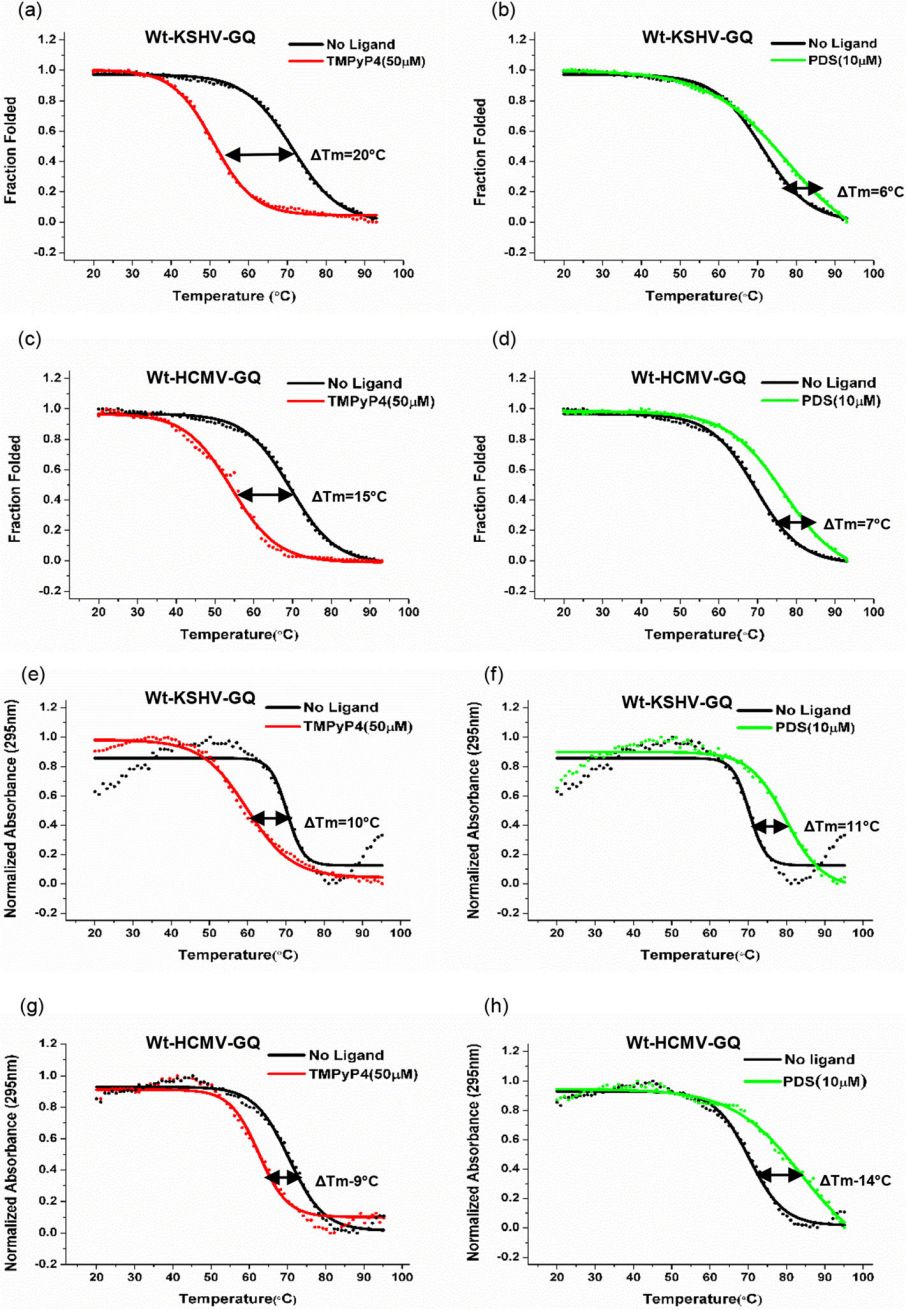
CD and UV melting curves demonstrating changes in thermal stability upon interaction with G-quadruplex ligands. (**a**)-(**d**) CD melting and (**e**)-(**h**) UV melting analyses of the Wt-KSHV-GQ and Wt-HCMV-GQ oligonucleotides under the effect of G-quadruplex ligands shows the destabilizing effect of TMPyP4 (50 μM) and stabilizing effect of PDS (10 μM) on the GQs under study. CD melting experiments were performed at a fixed wavelength of 262 nm. ΔTm is defined as the difference between the Tm of the PQS oligonucleotide in the presence and absence of ligand (i.e. TMPyP4 or Pyridostatin)

It has been demonstrated that the cationic porphyrin TMPyP4 and small molecule compound Pyridostatin (PDS) can bind to G-quadruplex strucures and either stabilize or destabilize them [17, 66, 67]. We performed CD melting experiments to determine melting temperatures (Tm) for Wt-KSHV-GQ and Wt-HCMV-GQ, respectively in the presence of TMPyP4 and PDS. The spectra shows that TMPyP4 destabilizes both Wt-KSHV-GQ and Wt-HCMV-GQ; on the other hand, PDS stabilizes both GQ motifs effectively (Fig. 6a-d). Our UV melting studies confirm our findings in CD melting (i.e. destabilizing effect of TMPyP4 and stabilizing effect of PDS on both GQ-motifs; Fig. 6e-h).

### G-quadruplexes regulate miRNA promoter activity in human herpesviruses

**Fig. 7:**
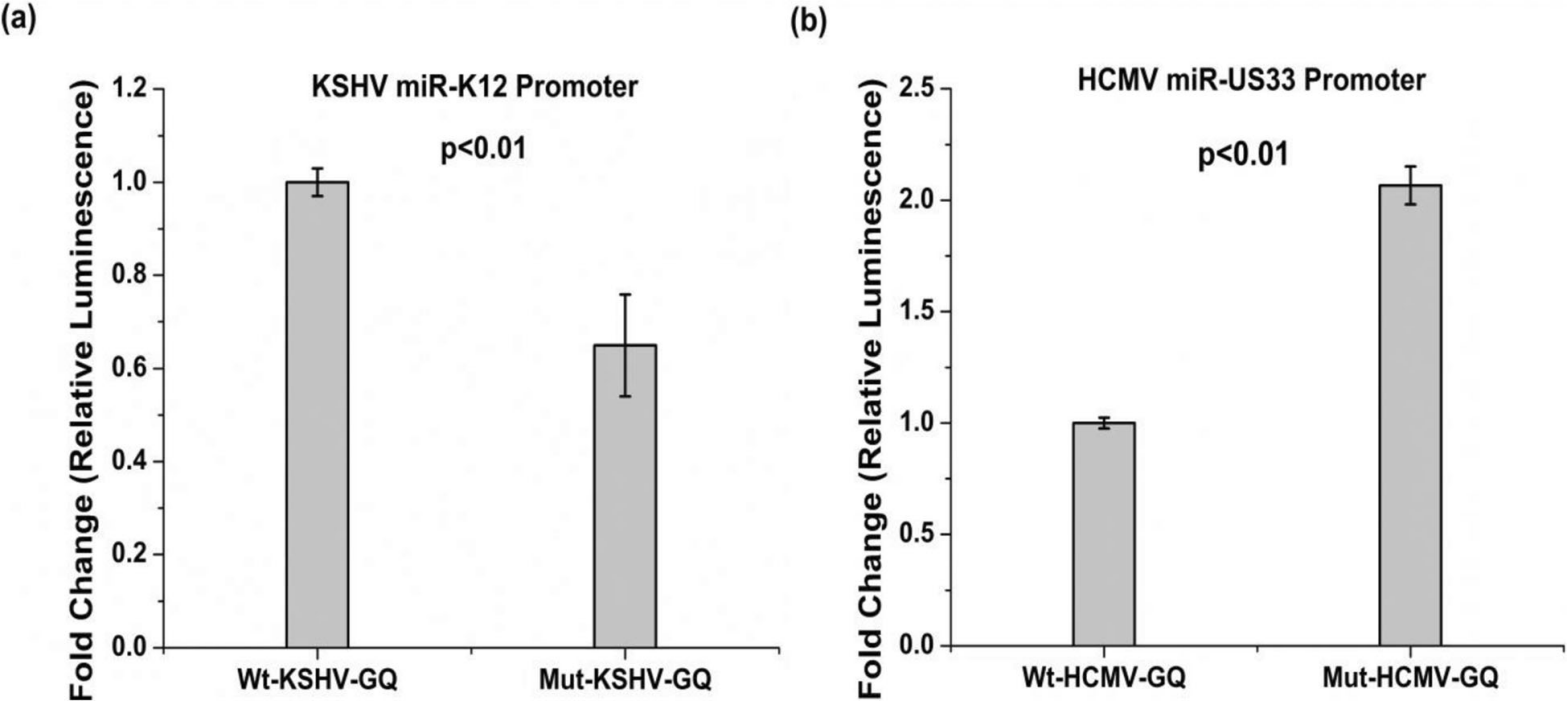
G-quadruplexes modulate promoter activity of the KSHV miR-K12 cluster and the HCMV miR-US33 promoters. Bar graphs showing promoter activity as measured by firefly luciferase levels normalized with renilla luciferase levels (transfection control). The relative luciferase units (RLU) values of the mutant constructs (Mut-KSHV-GQ and Mut-HCMV-GQ) were nomarlized to that of the respective wild-type constructs (Wt-KSHV-GQ and Wt—HCMV-GQ). (**a**) The Wt-KSHV-GQ construct (wild-type KSHV miR-K12 cluster promoter with an intact G-quadruplex) has significantly higher promoter activity as compared to that of the Mut-KSHV-GQ construct (contains mutations disrupting the G-quadruplex in the KSHV miR-K12 cluster promoter) (**b**) The Wt-HCMV-GQ construct (wild-type HCMV miR-US33 promoter with an intact G-quadruplex) has significantly reduced promoter activity compared to that of the Mut-HCMV-GQ construct (contains mutations disrupting the G-quadruplex in the HCMV miR-US33 promoter). Data are depicted as mean ± SD with *n* = 4 replicates

To examine the role of the respective GQs on miRNA promoter activity, wild type KSHV-GQ and HCMV-GQ promoters were cloned into firefly luciferase reporter vector (pGL3-basic). Mutant constructs were designed where the central guanines in each G-tract were substituted with adenines (please see Fig. 4a and the methods section for details). The mutations were incorporated with a motive of disrupting G-quadruplex formation. HEK293T cells were co-transfected with firefly luciferase constructs (i.e. the wild-type or the mutant constructs) or empty pGL3 basic vector along with internal control pRL-TK vector (a renilla luciferase reporter construct with a thymidine kinase promoter). Interestingly, the Mut-KSHV-GQ construct (i.e. the promoter of the the KSHV miR-K12 cluster with mutations disrupting the GQ) showed ~ 40% reduction in promoter activity compared to the Wt-KSHV-GQ contruct (i.e the wild-type promoter of the the KSHV miR-K12 cluster with an intact GQ; Fig. 7a). In contrast, the Mut-HCMV-GQ construct (i.e the promoter of the HCMV miR-US33 with mutations disrupting the GQ) was associated with approximately 2-fold increase in HCMV miR-US33 promoter activity compared to the Wt-HCMV-GQ construct (i.e the wild-type promoter of the HCMV miR-US33 with an intact GQ; Fig. 7b). In other words, these findings indicate that the presence of the G-quadruplex in the KSHV miR-K12 cluster promoter is associated with increased promoter activity and while that in the HCMV miR-US33 promoter is associated with reduced promoter activity.

We also analyzed the promoter activity of Wt-KSHV-GQ and Wt-HCMV-GQ promoters with two G-quadruplex ligands namely, TMPyP4 and PDS. Both ligands (TMPyP4 or Pyridostatin) were first tested for toxicity on HEK293T cells (Additional file 1; Figure S1). We then transfected the wild-type constructs in HEK293T cells and treated them with increasing concentrations of TMPyP4 or PDS (added 2 h post transfection). The lucifearse assay was performed after 24 h of ligand exposure. With increasing concentrations of TMPyP4 (which destabilized the GQ in KSHV promoter), the Wt-KSHV-GQ promoter (containing an intact GQ) showed marginal decrease in promoter activity uptil 20 μM concentration, however Wt-KSHV-GQ promoter activity drastically reduced at 50 μM TMPyP4 concentration (Additional file 1; Figure S2a). On the other hand, the Wt-KSHV-GQ promoter activity progressively increased with increasing concentrations of PDS (which stabilized the GQ in the KSHV promoter; Additional file 1; Figure S2b). The promoter activity of the HCMV miR-US33 promoter increased (Additional file 1; Figure S2c and S2d) with increasing concentration of TMPyP4 (which destabilized the GQ in the HCMV promoter), and decreased with increasing concentration of PDS (which stabilized the GQ in the HCMV promoter). These results indicate that the G-quadruplex in the KSHV miR-K12 cluster promoter enhances promoter activity and the G-quadruplex in the HCMV miR-US33 promoter inhibits promoter activity.

**Fig. 8:**
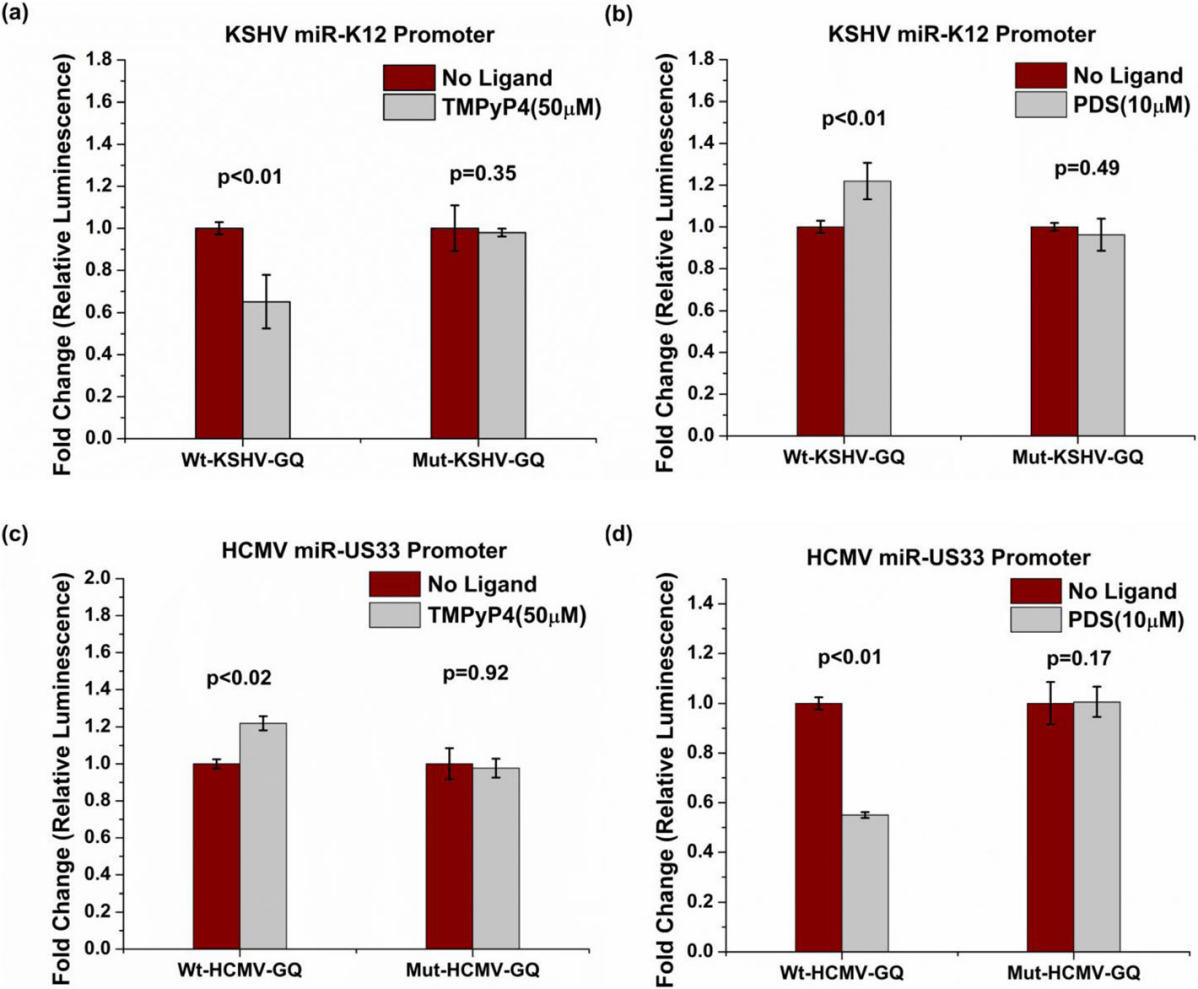
G-quadruplex ligands modulate promoter activity of KSHV and HCMV miRNAs for wild-type contructs but not mutant constructs. Bar graphs showing promoter activity as measured by firefly luciferase levels normalized with renilla luciferase levels (transfection control). The relative luciferase units (RLU) values of the reporter constructs in the presence of the ligand were nomarlized to that of the respective reporter constructs in the absence of the ligand. (**a**) The addition of TMPyP4 (50 μM) was associated with significant reduction in promoter activity of Wt-KSHV-GQ promoter, however (**b**) the addition of PDS (10 μM), led to a significant increase in promoter activity of the Wt-KSHV-GQ promoter. (**c**) Conversely, TMPyP4 (50 μM) significantly enhanced Wt-HCMV-GQ promoter activity while (**d**) PDS (10 μM) inhibited the Wt-HCMV-GQ promoter activity. It is clear from panel (**a**) through (**d**) that neither TMPyP4 nor PDS alter the promoter activity the mutant reporter constructs i.e. Mut-KSHV-GQ and Mut-HCMV-GQ; both containing G-quadruplex disrupting mutations. These findings ascertain a role for DNA secondary strucutures in modulating promoter activity of KSHV and HCMV miRNAs. Experiments were performed in triplicates and mean values ±SD were plotted

To ascertain that the observed differences between the wild-type and the respective mutant constructs is not due to primary sequence changes in the mutant, we analyzed the difference in promoter activity of the Wt and Mut contructs in the presence of the 50 μM TMPyP4 (which destabilized the GQ in both promoters) or 10 μM PDS (which stabilized the GQ in both promoters). The addition of TMPyP4 which destabilized the GQ in KSHV miR-K12 cluster promoter was associated with a reduction in promoter activity of the Wt-KSHV-GQ promoter but did not affect the promoter activity of the Mut-KSHV-GQ (Fig. 8a). The addition of PDS which stabilized the GQ in KSHV miR-K12 cluster promoter was associated with an increase in the promoter activity of the Wt-KSHV-GQ promoter but did not affect the promoter activity of the Mut-KSHV-GQ (Fig. 8b). This finding is in keeping with the positive regulatory role for the GQ upstream of KSHV miR-K12 cluster. In addition, neither of the ligands significantly affected the promoter activity of the Mut-KSHV-GQ construct, indicating that the reduced promoter activity observed for this construct (as compared to the wild-type construct) is associated with DNA secondary structure and not with primary sequence changes. Similarly, the addition of TMPyP4 or PDS altered the promoter activity of the Wt-HCMV-GQ construct (with an intact GQ in the promoter) but not that of the Mut-HCMV-GQ (with mutations disrupting the GQ); this finding reiterates a negative regulatory role for the GQ in the HCMV miR-US33 promoter (Fig. 8c and d). In addition, these results also ascertain that the increased promoter activity in the Mut-HCMV-GQ construct is due to DNA secondary structures and is not associated with primary sequence changes.

## Discussion

It is well-documented that viral miRNAs alter viral as well as host gene expression to their advantage; this eventually helps the virus to survive and replicate inside the host. In addition, latency is an important feature of herpesvirus biology, associated with miRNA-mediated regulation of gene expression which allows the virus to escape immune surveillance [68, 69]. These observations suggest that miRNAs play a crucial role throughout the virus life cycle. Our findings support the notion of GQ-mediated regulation of herpesvirus miRNAs.

In sum, our findings suggest (a) the enrichment of G-quadruplex motifs in the proximal regions (1-200 bp) of herpesvirus-encoded miRNAs (b) this enrichment of G-quadruplexes in the promoters of herpesvirus-encoded miRNAs is not a random event associated with high G + C content of herpesvirus genomes (c) extensive biophysical analyses of the PQS in the KSHV miR-K12 cluster promoter and in the HCMV miR-US33 promoter confirms the formation of intramolecular G-quadruplexes in vitro (d) reporter assays using mutants with disrupted G-quadruplexes and with G-quadruplex interacting ligands indicate a positive regulatory role for the G-quadruplex in the KSHV miR-K12 cluster promoter and a negative regulatory role for that in the HCMV miR-US33 promoter. The virus miRNAs studied here are critical for maintenance of viral latency. MicroRNAs from the KSHV miR-K12 cluster directly inhibit the expression of KSHV RTA (Replication and Transcription Activator), which is a key modulator of virus as well as host gene expression [53]. Also, HCMV miR-US33 is known to suppress HCMV replication by inhibiting viral *US29* gene and host *STX3* gene expression [48, 60]. The pervasiveness of G-quadruplexes in the proximal promoter regions of these herpesvirus-encoded miRNAs, their ability to form G-quadruplex structures in vitro and their role in modulating promoter activity suggests that these DNA secondary structures represent a novel regulatory element of herpesvirus-encoded miRNAs.

To the best of our knowledge, this is the first report to elucidate the presence of G-quadruplex motifs in regulatory regions of herpesvirus-encoded miRNAs. These findings have important implications to our current understanding of herpesvirus biology.

## Conclusions

Taken together our findings convincingly demonstrate a positive regulatory role for the GQ in the KSHV miR-K12 cluster promoter and a negative regulatory role for the GQ in the HCMV miR-US33 promoter. The GQ-mediated modulation of herpesvirus-encoded miRNAs in turn may regulate target mRNA levels (virus or host encoded). In sum, this work highlights G-quadruplex-mediated regulation of herpesvirus-encoded miRNAs.

## Methods

### Retrieval of sequences

All virus miRNA sequences (mature and precursor miRNAs) were obtained from the microRNA database miRBase (v22.1) [34]. Full-length herpesvirus genomes were obtained from NCBI GenBank and ViPR database (http://www.viprbrc.org) [70].

The upstream sequences of precursor miRNAs of all virus strains under study were obtained as follows. First, if a precursor miRNA overlaps with another gene and were unidirectional, the 1000 bp region upstream of the concerned gene was obtained; on the other hand, if a precursor miRNA and the gene were convergent, the 1000 bp region upstream of the precursor miRNA was obtained. Second, if precursor microRNAs were known to be intergenic, the 1000 bp region upstream of the precursor miRNA was retrieved.

### PQS mapping

The retrieved upstream sequences were analyzed using Quadparser (a computer algorithm) [61] to identify PQS with parameters (minimum G-tetrad-3 and loop length- 1-15). PQS density was defined as the total number of non-overlapping PQS predicted per kilo base of the sequence analyzed. Average PQS densities were computed for analysis.

### Randomization of sequences

In order to determine whether the occurrence of PQS motifs in the retrieved sequences is a random/non-random event, the selected sequences were shuffled while preserving the dinucleotide frequencies. This was achieved by performing a dinucleotide shuffle of the selected sequences (without changing the overall nucleotide composition). To do so, the base pairs were selected by randomly generated base numbers and the Eulerian walk method was employed while satisfying the constraint of keeping the number of dinucleotides constant before and after shuffling. The shuffling were performed 5 times. The python script (Additional file 2) used for dinucleotide shuffling of the 1000 bp sequences under study, is based on the freely available ‘uShuffle’ program script [71] with some modifications to facilitate easy analysis of the necessary parameters. Additional details are provided in a ‘Readme’ text file. Average PQS densities were mapped in the randomized sequences generated and were compared to that in the native sequences.

### PQS conservation analysis

Upstream 1 kb sequences of herpesvirus miRNA promoters possessing at least 1 PQS motif (identified in the full-length virus sequences) were retrieved and studied for conservation analysis by performing multiple sequence alignment. Sequences for full length virus strains were downloaded from NCBI GenBank and ViPR database (http://www.viprbrc.org) [70]. Accession numbers of all sequences analyzed are mentioned in Additional file 1; Table S4. PQS motifs with intact Gs in the consecutive G-tetrads were considered conserved. Loop sequences with variable length and composition were not taken into account for conservation analysis.

### Circular Dichroism spectroscopy and melting studies

CD studies were performed on a Chirascan circular dichroism spectrometer (Applied Photophysics Limited, UK). The sequences of the 2 PQS-motifs used (wild type and mutant) are listed in Additional file 1; Table S2. The oligonucleotides were purchased from Integrated DNA Technologies (IDT) for all biophysical experiments. Oligonucleotides (10 μM) were dissolved in 10 mM sodium cacodylate buffer (pH -7.5) along with 100 mM potassium chloride (KCl). The samples were heated at 95 °C for 5 min and slowly cooled to room temperature. A quartz cuvette (1 mm path length) was used for recording of spectra at the wavelength range (220–320 nm) with a 1 nm bandwidth, 1 nm step size and time of 1 s per point at 20 °C. CD melting was performed at a fixed concentration of oligonucleotides (10 μM), either with or without a fixed concentration of G-quadruplex ligands TMPyP4 and pyridostatin (PDS). The data was recorded at a ramp rate of 1 °C/minute over a range of 20–93 °C. A buffer baseline was recorded and subtracted from the sample spectra. Tm (melting temperature) was calculated by the first derivative method. Final analysis of the data was conducted using Origin 9.1 (Origin Lab Corp.).

### NMR spectroscopy

The oligonucleotide samples were heated at 95 °C for 5 min and slowly cooled to room temperature. The NMR sample contained 300 μM oligonucleotides in 20 mM potassium phosphate buffer (pH 7.0), 100 mM KCl and 10% D_2_O (v/v). 1D ^1^H NMR spectra were recorded using Bruker Avance III spectrometer equipped with cryogenic 5 mm TCI triple-resonance probe, operating at a field strength of 500 MHz. The spectra were recorded at 20 °C using Topspin 3.5 (Bruker AG). Data processing and analysis were performed with Topspin 4.6 software (Bruker AG).

### Polyacrylamide gel electrophoresis

Oligonucleotides were prepared at 10 μM concentration in Tris-EDTA buffer (pH -7.0) and 100 mM KCl. The samples were heated at 95 °C for 5 min and slowly cooled to room temperature before loading. Native and denaturing polyacrylamide gels were prepared in 1× Tris-borate EDTA (TBE) buffer. 7 M urea was used as a denaturant to prepare denaturing polyacrylamide gel. Gels were run in 0.5× TBE with 50 mM KCl.

### UV melting studies

A Cary 100 Bio UV-Vis double-beam spectrophotometer (Agilent Technologies) equipped with a multi-cell holder attached to a Peltier controller was used to perform UV melting experiments. Oligonucleotides at a concentration of 4 μM were mixed with 10 mM sodium cacodylate (pH -7.5) and 100 mM KCl. For ligand studies, fixed concentrations of TMPyP4 and PDS were used. The melting curves were recorded at 295 nm both ways (melting and annealing) between 20 °C and 95 °C with a ramp rate of 1 °C/min. Origin 9.1 (Origin Lab Corp.) was used to analyze and plot melting curves.

### Luciferase constructs

The native promoter of KSHV miR-K12 cluster was amplified by PCR from KSHV JSC-1 genomic DNA which was kindly provided by Dr. Tathagata Choudhuri (Visva Bharati University, West Bengal, India), while HCMV miR-US33 promoter region was commercially synthesized by Life Technologies Corp. The wild type and mutant promoters were cloned in pGL3-basic vector (Promega) upstream of firefly luciferase coding sequence using appropriate primers listed in Additional file 1; Table S3. The plasmid constructs were extracted using QIAprep Spin Miniprep Kit (Qiagen) and confirmed by sequencing.

### Cell proliferation assay (MTT)

Cell proliferation assay was performed in 96 well plate by incubating HEK293T cells (seeding density = 1 × 10^4^ cells/well) in the presence of multiple doses of TMPyP4 (Sigma) or Pyridostatin (Sigma). After 24 h, cells were exposed to MTT (3-(4,5-Dimethylthiazol 2-yl)-2,5-diphenyltetrazolium bromide) (Sigma) reagent for 1 h. The medium was replaced with 100 μl dimethylsulfoxide (DMSO) and optical density measured at 570 nm (Additional file 1, Figure S1).

### Luciferase reporter assay

HEK293T cells (procured from NCCS, Pune, India) were maintained in Dulbecco’s modified medium (Invitrogen) supplemented with 10% fetal bovine serum and were incubated at 37 °C and with 5% CO_2_. HEK293T cells were seeded in 24-well plates at a density of 5 × 10^4^ cells/well 24 h prior to transfection. The luciferase reporter constructs (wild-type or mutant; 500 ng each) and 20 ng of pRL-TK (25:1 ratio) were co-transfected using PEI (polyethylenimine) into HEK293T cells in 24-well plates. For ligand studies, G-quadruplex ligands TMPyP4 and Pyridostatin were added 2 h after transfection at the appropriate concentration. Both ligands were used in the absence of light. At 24 h post-transfection, cell lysates were prepared using passive lysis buffer. Luciferase assays were performed using a dual luciferase reporter assay system according to the manufacturer’s protocols (Promega) with MicroBeta2 Microplate Scintillation Counter (Perkin Elmer). Firefly luciferase activity was normalized to renilla luciferase activity. Three independent experiments were done in triplicates.

### Data analyses

Data was plotted as mean values ± SD in at least three distinct experiments. The statistically significant difference was defined as *P* < 0.01 calculated using Student’s t-test unless mentioned otherwise. Figure 1 and Fig. 3 were made using Microsoft Powerpoint. R software was used to generate violin plot (Fig. 2a). Origin 9.1 (Origin Lab Corp.) was used to plot melt curves and bar graphs.

## Supplementary information

**Additional file 1 Table S1.** List of herpesvirus encoded miRNAs. **Table S2.** Name and sequence of oligonucleotides**. Table S3.** List of primers used to make luciferase constructs. **Table S4.** List of virus strains. **Table S5.** List of PQS found upstream of herpesvirus encoded miRNAs. **Figure S1.** MTT assay for cell viability in HEK293T cells for G-quadruplex binding ligands namely (a) TMPyP4 and (b) PDS. **Figure S2.** Effect of varying doses of TMPyP4 and PDS on Wt-KSHV-GQ and Wt-HCMV-GQ promoter activity.

**Additional file 2.** Python script for dinucleotide shuffling.

## Data Availability

The datasets used and/or analysed during the current study are available from the corresponding author on reasonable request.

## Abbreviations

GQ: G-Quadruplex
KSHV: Kaposi's sarcoma-associated herpesvirus
HCMV: Human cytomegalovirus
PQS: Putative quadruplex forming sequence
