# Supplementary material for: Analysis of G-quadruplexes upstream of herpesvirus miRNAs: evidence of G-quadruplex mediated regulation of KSHV miR-K12–1-9,11 cluster and HCMV miR-US33
Source: BMC Mol Cell Biol. 2020 Sep 24;21:67. doi: 10.1186/s12860-020-00306-w (PMC7513282; doi:10.1186/s12860-020-00306-w)
Supplement: Supplementary file 1 — Additional file 1 Table S1. List of herpesvirus encoded miRNAs. Table S2. Name and sequence of oligonucleotides. Table S3. List of primers used to make luciferase constructs. Table S4. List of virus strains. Table S5. List of PQS found upstream of herpesvirus encoded miRNAs. Figure S1. MTT assay for cell viability in HEK293T cells for G-quadruplex binding ligands namely (a) TMPyP4 and (b) PDS. Figure S2. Effect of varying doses of TMPyP4 and PDS on Wt-KSHV-GQ and Wt-HCMV-GQ promoter activity. [file 12860_2020_306_MOESM1_ESM.zip › 12860_2020_306_MOESM1_ESM/Additional File 1.docx]

**Table S1. List of all herpesvirus encoded miRNAs**

**Table S2. Name and sequence of oligonucleotides used for biophysical experiments.**


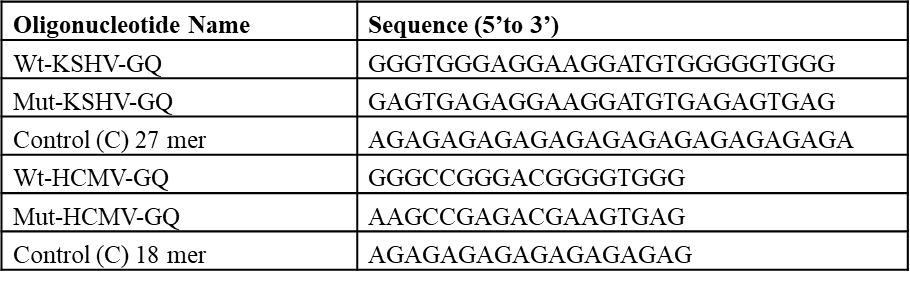


**Table S3. List of primers used to make luciferase constructs.**


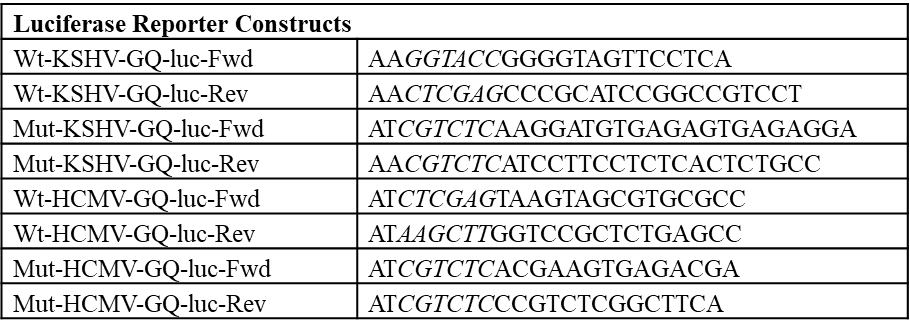


**Table S4. List of virus strains.**

| **Virus** | **Accession Number** |
| --- | --- |
| **HHV-1 (HSV-1)** | AB618031, FJ593289, GU734771, GU734772, JN555585, JQ673480, JQ780693, KF498959, NC_001806, X14112, KJ847330, CS176883 |
| **HHV-2 (HSV-2)** | JN561323, NC_001798, Z86099, KR135322, KR135323, KR135324, KR135325, KR135326, KR135327, KR135328, KR135329, KR135299, KR135300, KR135301, KR135302, KR135303, KR135304, KR135305, KR135306, KR135307, KR135315, KR135316, KR135317, KR135318, KR135319, KR135303, KR135298, KR135308, KR135309, KR135310, KR135311, KR135312, KR135313, KR135314, KR135321, KR135330, KR135331, KR135320, KF781518 |
| **HHV-5 (HCMV)** | AC146851, AC146904, AC146905, AC146906, AC146907, AC146999, AY315197, AY446894, BK000394, EF999921, FJ527563, FJ616285, GQ121041, GQ221973, GQ221974, GQ221975, GQ396662, GQ396663, GQ466044, GU179001, GU179288, GU179289, GU179290, GU937742, GU980198, HQ380895, JX512197, JX512198, JX512199, JX512200, JX512201, JX512202, JX512203, JX512204, JX512205, JX512206, JX512207, JX512208, KF021605, KF297339, KF493876, KF493877, KJ361971, KJ426589, KM192298, KM192299, KM192300, KM192301, KM192302, NC_006273, X17403 |
| **HHV-8 (KSHV)** | CS208161, CS208162, CS208163, JQ619843, AF148805, NC_009333, GQ994935, HQ404500, KF703446, U75698.1 |
| **BHV-1** | NC_001847.1, KU198480.1, AJ004801.1, KM258880, KM258881, KM258882, KM258883 |
| **BHV-5** | NC_005261.2, AY261359.1 |
| **HBV** | AF533768, NC_004812, KJ566591 |
| **RLCV** | NC_006146.1, AY037858.1, KP676001.1 |
| **MCMV** | GU305914, NC_004065, U68299, AM886412, EU579861, EU579859, EU579860, HE610451, HE610452, HE610453, HE610454, HE610455, HE610456 |
| **HHV-6B** | NC_000898, AF157706.1 |
| **MDV-2** | AB049735, NC_002577, HQ840738, AB024414 |

**Table S5. List of PQS found upstream of herpesvirus encoded miRNAs.**

*Primary strand refers to the sequence submitted in NCBI.

**Figure S1.**

**
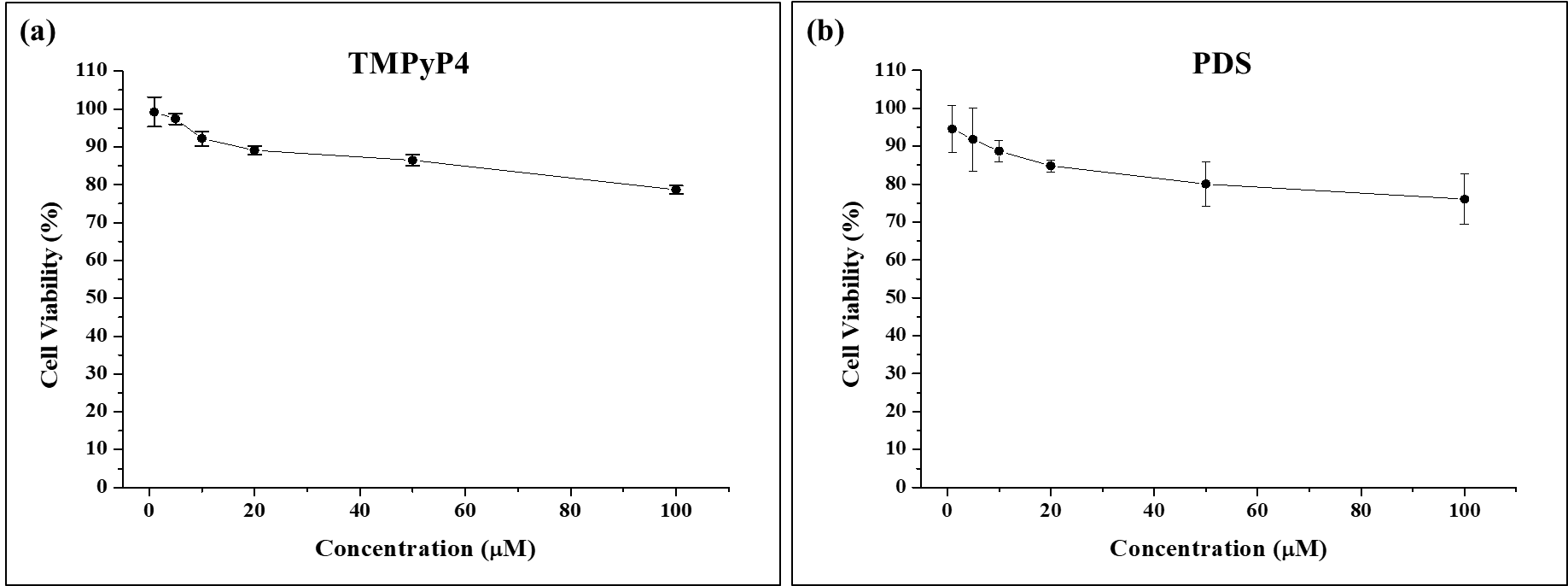
**

**Figure S1. MTT assay for cell viability in HEK293T cells for G-quadruplex binding ligands namely (a) TMPyP4 and (b) PDS.**

**Figure S2.**

**
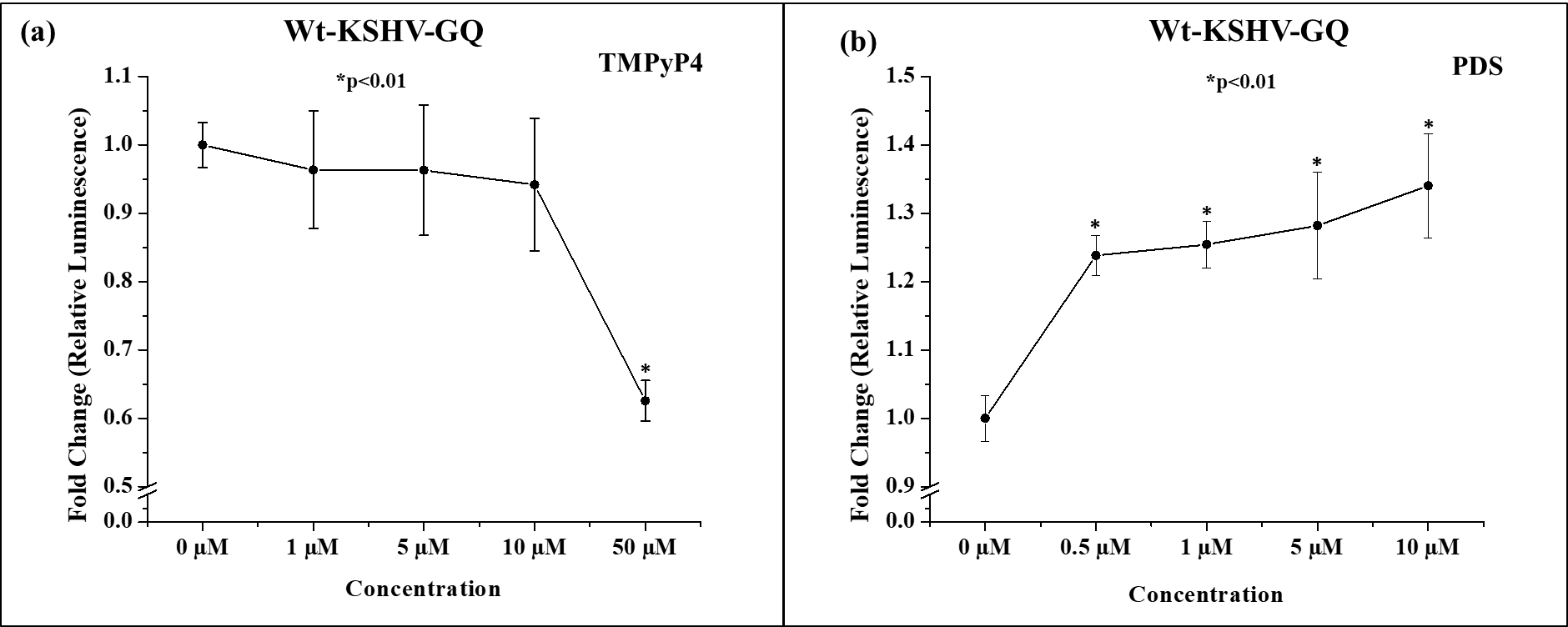
**
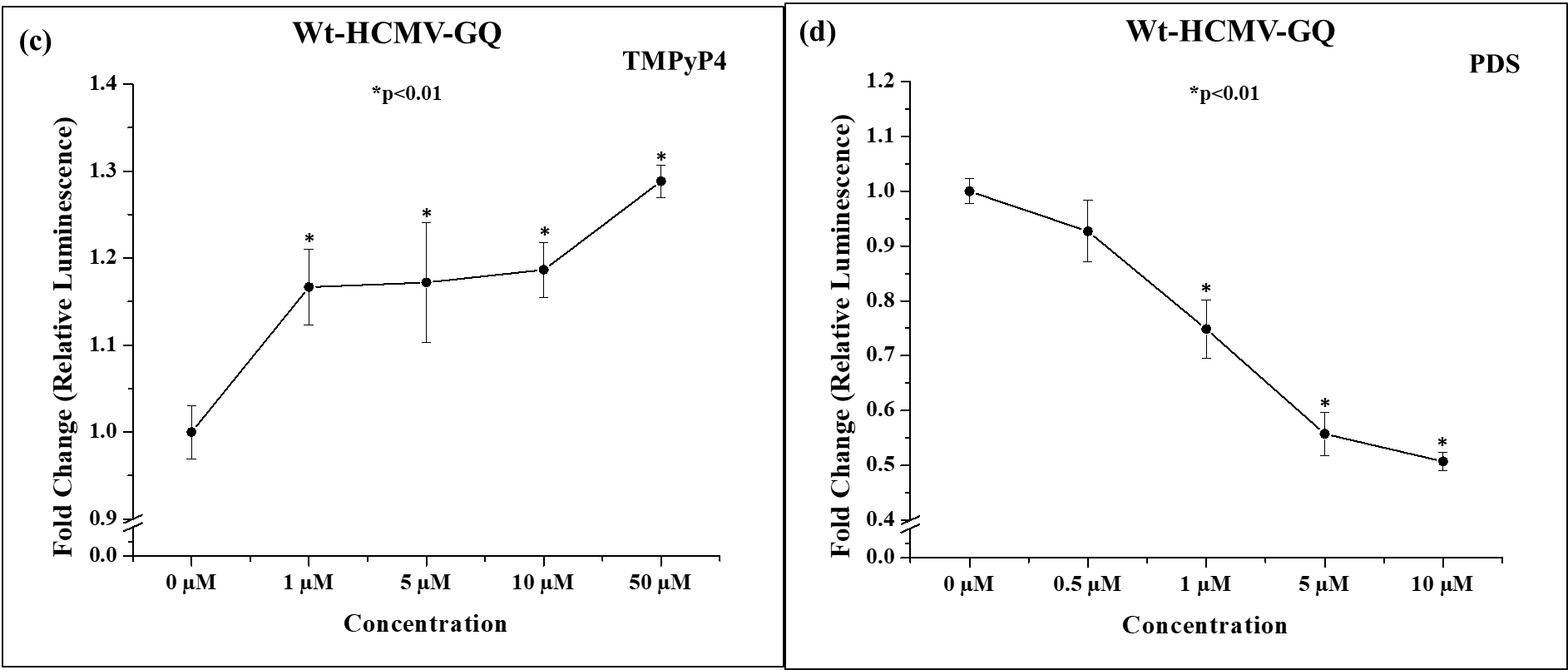


**Figure S2. Effect of varying doses of TMPyP4 and PDS on Wt-KSHV-GQ and Wt-HCMV-GQ promoter activity.**

(a) and (b) The promoter activity of Wt-KSHV-GQ promoter decreases with increasing concentrations of TMPyP4 and increases with increasing concentrations of PDS. (c) and (d) On the other hand, promoter activity of Wt-HCMV-GQ promoter increases with increasing concentrations of TMPyP4 and decreases with increasing concentrations of PDS.
